# Synthesis and Cytotoxicity of Cyanoborane Adducts of $N^{6}$-Benzoyladenine and 6-Triphenylphosphonylpurine

**DOI:** 10.1155/MBD.2002.19

**Published:** 2002

**Authors:** Tanya C. Scarlett, Richard W. Durham, Iris H. Hall, Richard J. Crosswicks, Joshua D. Berkowitz, Bruce S. Burnham

**Affiliations:** 1 Division of Medicinal Chemistry and Natural Products School of Pharmacy University of North Carolina Chapel Hill 27599-7360 USA; 2 Department of Chemistry and Biochemistry Rider University 2083 Lawrenceville Rd. Lawrenceville NJ 08648 USA

## Abstract

$N^{6}$-Benzoyladenine-cyanoborane (2), and 6-triphenylphosphonylpurine-cyanoborane (3) were selected for investigation of cytotoxicity in murine and human tumor cell lines, effects on human HL-60 leukemic metabolism and DNA strand scission to determine the feasibility of these compounds as clinical antineoplastic agents. Compounds 2 and 3 both showed effective cytotoxicity based on ED_50_ values less than 4 μg/ml for L1210, P388, HL-60, Tmolt_3_, HUT-78, HeLa-S_3_ uterine, ileum HCT-8, and liver Hepe-2. Compound 2 had activity against ovary 1-A9, while compound 3 was only active against prostate PL and glioma UM. Neither compound was active against the growth of lung 549, breast MCF-7, osteosarcoma HSO, melanoma SK2, KB nasopharynx, and THP-1 acute monocytic leukemia. In mode of action studies in human leukemia HL-60 cells, both compounds demonstrated inhibition of DNA and protein syntheses after 60 min at 100 μM. These compounds inhibited RNA synthesis to a lesser extent. The utilization of the DNA template was suppressed by the compounds as determined by inhibition of the activities of DNA polymerase α, m-RNA polymerase, r-RNA polymerase and t-RNA polymerase, which would cause adequate inhibition of the synthesis of both DNA and RNA. Both compounds markedly inhibited dihydrofolate reductase activity, especially in compound 2. The compounds appeared to have caused cross-linking of the DNA strands after 24 hr at 100 μM in HL-60 cells, which was consistent with the observed increased in ct-DNA viscosity after 24 hr at 100 μM. The compounds had no inhibitory effects on DNA topoisomerase I and II activities or DNA-protein linked breaks. Neither compound interacted with the DNA molecule itself through alkylation of the nucleotide bases nor caused DNA interculation between base pairs. Overall, these antineoplastic agents caused reduction of DNA and protein replication, which would lead to killing of cancer cells.


### Synthesis and Cytotoxicity of Cyanoborane Adducts of $N^6$-Benzoyladenine and 6-Triphenylphosphonylpurine

Tanya C. Scarlett, Richard W. Durham, Jr and Iris H. Hall
*Division of Medicinal Chemistry and Natural Products,*
*School of Pharmacy, University of North Carolina, Chapel Hill 27599-7360, USA*

Richard J. Crosswicks, Joshua D. Berkowitz and Bruce S. Burnham
*Department of Chemistry and Biochemistry, Rider University, 2083 Lawrenceville Rd.,*
*Lawrenceville, NJ 08648, USA*

### ABSTRACT

$N^6$-Benzoyladenine-cyanoborane (2), and 6-triphenylphosphonylpurine-cyanoborane (3) were selected for investigation of cytotoxicity in murine and human tumor cell lines, effects on human HL-60 leukemic metabolism and DNA strand scission to determine the feasibility of these compounds as clinical antineoplastic agents. Compounds 2 and 3 both showed effective cytotoxicity based on $ED_{50}$ values less than 4 μg/ml for L1210, P388, HL-60, $Tmolt_3$, HUT-78, HeLa-$S^3$ uterine, ileum HCT-8, and liver Hepe-2. Compound 2 had activity against ovary 1-A9, while compound 3 was only active against prostate PL and glioma UM. Neither compound was active against the growth of lung 549, breast MCF-7, osteosarcoma HSO, melanoma SK2, KB nasopharynx, and THP-1 acute monocytic leukemia. In mode of action studies in human leukemia HL-60 cells, both compounds demonstrated inhibition of DNA and protein syntheses after 60 min at 100 μM. These compounds inhibited RNA synthesis to a lesser extent. The utilization of the DNA template was suppressed by the compounds as determined by inhibition of the activities of DNA polymerase α, m-RNA polymerase, r-RNA polymerase and t-RNA polymerase, which would cause adequate inhibition of the synthesis of both DNA and RNA. Both compounds markedly inhibited dihydrofolate reductase activity, especially in compound 2. The compounds appeared to have caused cross-linking of the DNA strands after 24 hr at 100 μM in HL-60 cells, which was consistent with the observed increased in ct-DNA viscosity after 24 hr at 100 μM. The compounds had no inhibitory effects on DNA topoisomerase I and II activities or DNA-protein linked breaks. Neither compound interacted with the DNA molecule itself through alkylation of the nucleotide bases nor caused DNA interculation between base pairs. Overall, these anti-neoplastic agents caused reduction of DNA and protein replication, which would lead to killing of cancer cells.

### INTRODUCTION:

Potent anti-neoplastic activity has been demonstrated for cyanoborane derivatives of adenosine, guanosine, inosine, and cytidine /1-8/. Base substituted boronated nucleosides and phosphate-modified nucleotides have been reported to be even more potent in suppressing the growth of murine and human cancer cells /2/. A common feature of all of these derivatives was that they effectively suppressed DNA synthesis and the activities of enzymes involved in the nucleic acid metabolism. Selected compounds demonstrated DNA strand scission with inhibition of DNA topoisomerase II activity. Based on these previous studies, $N^6$-benzoyladenine-cyanoborane (**2**), 6-triphenyl-phosphonylpurine-cyanoborane (**3**) were selected for investigation in human leukemia HL-60 cells for cytotoxicity, effects on metabolic events, and DNA strand scission to determine the feasibility of these compounds as clinical antineoplastic agents.

### METHODS

The cyanoborane adducts of the substituted purines were prepared *via* a Lewis acid exchange reaction /9/, by using excess (*e.g.*, 4 molar equivalents) triphenylphosphine-cyanoborane (1) and the amine (purine) in dry dimethylformamide (DMF) at 55-70°C under nitrogen atmosphere (Scheme 2). In the Lewis acid exchange reaction, a weakly basic or bulky amine or phosphine, as its substituted borane

$$(C_6H_5)_3P{\cdot}HBr + NaBH_3CN \xrightarrow[N_2(g)]{\text{dry THF, Reflux}} \underset{\mathbf{1}}{(C_6H_5)_3P{:}BH_2CN} + HBr + H_2$$

**Scheme 1** : Synthesis of the Lewis acid exchange reagent, triphenylphosphine-cyanoborane (**1**).

adduct (*e.g.*, $Ph_3P{:}BH_2CN$), is exchanged for a more basic or less bulky amine, (*e.g.*, a substituted purine). These boron exchange reactions must be carried out under anhydrous conditions to avoid coordination of the cyanoborane to water and subsequent degradation to boric acid. The Lewis acid exchange reaction is a general route which has also been used in the preparation of other cyanoborane adducts of aliphatic /3, 9/, aromatic /3,8/, and heterocyclic /8,9/ amines, as well as their borane and carboxyborane adducts /9/. Triphenylphosphine-cyanoborane (1) was prepared as previously reported /9, 10/ (Scheme 1) by refluxing $Ph_3P\,HBr$ and $NaBH_3CN$ in dry THF under nitrogen atmosphere. The reaction of 6-chloropurine and triphenylphosphine-cyanoborane produced the unexpected product, 6-triphenylphosphonylpurine-cyanoborane (3). This resulted when the free triphenylphosphine, formed after the Lewis acid exchange, underwent aromatic nucleophilic substitution displacing the chlorine in the 6 position of the purine ring.

### Synthesis of Compounds

All chemicals and reagents were obtained from Aldrich Chemical Company (Milwaukee, WI) and used as received except for dry solvents which were dried and distilled using standard procedures /11/. TLC was performed using silica gel 60F 254 plates (silica gel on plastic, Aldrich Chemical Company). Melting points were obtained on a Thomas-Hoover Uni-melt apparatus (capillary method), and were uncorrected. IR spectra were obtained on a Perkin-Elmer 1600 FTIR spectrometer in a potassium chloride liquid cell in $CHCl_3$ or $CDCl_3$. NMR spectra were obtained on a 300 MHz Bruker Avance FT-NMR spectrometer using tetramethylsilane as an external standard for $^1H$ and $^{13}C$ spectra and $BF_3$:$OEt_2$ for $^{11}B$ spectra ($\delta$ = 0 ppm). Elemental analyses were performed by Quantitative Technologies, Inc. (Whitehouse, NJ).

### Preparation of Triphenylphosphine-cyanoborane (1):

To a mixture of 4.02 g (11.7 mmol) of triphenylphosphine hydrobromide and 40 mL of dry THF was added 0.93 g (14.8 mmol) of sodium cyanoborohydride. The suspension was stirred under $N_2$ (*g*) at reflux for 10 hr. The mixture was cooled to RT, filtered and the solid washed with THF. The filtrate and washings were combined and the solvents were removed under reduced pressure. The white solid was washed with cold water, then cold ethyl ether. After air drying, 3.1002 g (10.30 mmol) of pure white solid was obtained in 88% yield. TLC on silica gel in 97.5:2.5 dichloromethane:methanol showed a single spot, $R_f$ =0.65. mp = 172-173°C, IR ($CHCl_3$): $\nu$ = 2360 $cm^{-1}$ (B-H), 2196 $cm^{-1}$ (-CN). $^1H$ NMR ($CDCl_3$): $\delta$ = 7.45-7.62 (m, 15H, Ph), 1.30-2.79 (br.m., 2H, $BH_2$) ppm. $^{11}B$ NMR ($CDCl_3$): $\delta$ = -36.68 ppm (q, $J_{B,H}$ = 94.1 Hz). $^{31}P$ NMR ($CDCl_3$): $\delta$ = 12.46 ppm (d, $J_{P,B}$ = 86.95 Hz).

### Preparation of $N^6$-Benzoyladenine-cyanoborane (2)

To a solution of 10.01 g (33.23 mmol, 4 Eq.) of triphenylphosphine-cyanoborane in 30 mL of dry DMF was added 2.03 g (8.49 mmol) of $N^6$-benzoyladenine. The solution was stirred under $N_2$ (*g*) at 70°C for 10 days. The solution was cooled to RT, filtered and the solid washed with methanol. The filtrate and washings were combined and silica gel was added until all of the liquid was adsorbed. The solvents were removed under reduced pressure. The product was purified by column chromatography on silica gel using dichloromethane:methanol (95:5, $R_f$ = 0.69). A partial yield /12/ of 0.0858 g (0.309 mmol, 3.6%) of tan solid was obtained. mp = 149-152°C, IR ($CDCl_3$): $\nu$ = 2360 $cm^{-1}$ (B-H), 2255 $cm^{-1}$ (-CN). $^1H$ NMR ($CDCl_3$): $\delta$ = 7.64-7.71 (m, 4H, H3, H8, *o*-Ph), 7.45-7.55 (m, 3H, *m*- and *p*-Ph), 1.73 (br.s, 2H, $BH_2$) ppm. $^{13}C$ NMR ($CDCl_3$): $\delta$ = 133.64, 132.54, 132.49, 132.41, 132.36, 132.29, 128.96, 128.91, 128.80, 128.68 ppm. $^{11}B$ NMR ($CDCl_3$): $\delta$ = -33.20 ppm (br. peak). HRMS-FAB ($C_{13}H_{11}BN_6O$): $(M+H)^+$ = 279.1166 (theoretical), 279.0921 (found).

### Preparation of 6-triphenylphosphonylpurine-cyanoborane (3)

To a mixture of 15.76 g (52.34 mmol, 4 Eq.) of triphenylphosphine-cyanoborane in 30 mL of dry DMF was added 2.02 g (13.1 mmol) of 6-chloropurine. The mixture dissolved upon heating and was stirred under $N_2$ (*g*) at 70°C for 10 days. The solution was cooled to RT and the resulting suspension was filtered and the solid washed with methanol. The filtrate and washings were combined and silica gel was added until all of the liquid was adsorbed. The solvents were removed under reduced pressure. The product was purified by column chromatography on silica gel using dichloromethane:methanol (95:5, $R_f$ = 0.49). A partial yield /12/ of 0.1363 g (0.705 mmol, 5.4%) of yellow solid was obtained. mp = 209-212°C (dec), IR ($CHCl_3$): ν = 2393 $cm^{-1}$ (B-H). $^1H$ NMR ($CDCl_3$): δ = 9.18 (s, 1H, H3), 8.30 (s, 1H, H8), 1.82 (br.s, 2H, $BH_2$) ppm. $^{13}C$ NMR ($CDCl_3$): δ = 157.21, 151,70, 151.46, 138.09, 136.58, 119.09 ppm $^{11}B$ NMR ($CDCl_3$): δ = -21.4 ppm (br. peak). $^{31}P$ NMR ($CDCl_3$): δ = 16.88 ppm. MS ($C_{24}H_{19}BN_5P$): $(M-H)^+$ = 418. Elemental Analysis ($C_{24}H_{19}BN_5P$ 1/2$CH_3OH$): C: 67.61%, H: 4.86%, N:16.09% (theoretical), C: 67.47%, H: 4.62%, N:15.92% (found).

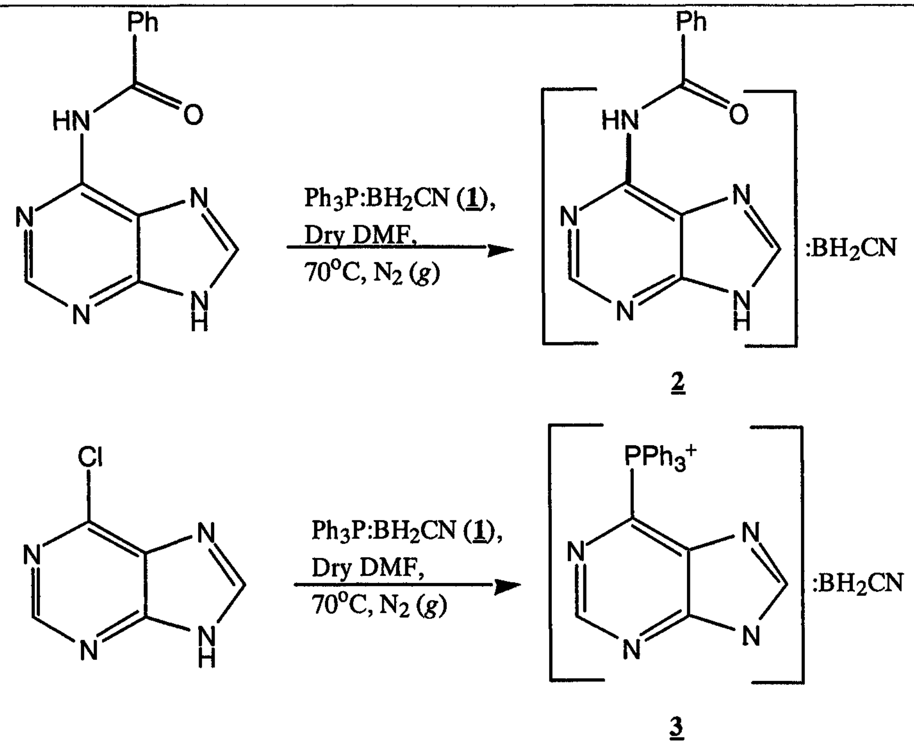

**Scheme 2**: Synthesis of the cyanoborane adducts of $N^6$-benzoyladenine (2) and 6-triphenylphosphonylpurine (3).

### Cytotoxicity

Compounds **2-3** were tested for cytotoxic activity by homogenizing the drugs as a 1 mg/mL solution in 0.05% Tween 80/$H_2O$. These solutions were sterilized by passing them through an acrodisc (0.45 μm). The following cell lines were maintained by literature techniques /14/: murine $L_{1210}$ lymphoid leukemia and P388 lymphocytic leukemia, human $Tmolt_3$ and $Tmolt_4$ acute lymphoblastic T cell leukemia, HL-60 leukemia, Hut-78 cutaneous lymphoma, THP-1 acute monocytic leukemia, HCT-8 ileocecal adenocarcinoma, liver Hepe-2, A-549 lung carcinoma, HSO osteosarcoma, KB epidermoid nasopharynx, HeLa-$S^3$ suspended cervical carcinoma, ovary 1-A9, SK-MEL-2 malignant, breast effusion MCF-7 and U-87-MG glioma.

Normal fibroblasts 1788 were also used to test cytotoxicity of the agents. The NCI protocol was used to assess the cytotoxicity of the test compounds and standards in each cell line. Values for cytotoxicity were expressed as $ED_{50}$ = μg/ml, i.e. the concentration of the compound inhibiting 50% of cell growth. $ED_{50}$ values were determined by the trypan blue exclusion technique /13/. A value of less than 4 μg/ml was required for significant activity of growth inhibition. Solid tumor cytotoxicity was determined utilizing crystal violet/MeOH and read at 580 nm (Molecular Devices) /14/.

### Incorporation Studies

Incorporation of labeled precursors into $^{3}$H-DNA, $^{3}$H-RNA and $^{3}$H-protein for $10^6$ HL-60 leukemia cells was obtained /15/ using a concentration range of 25, 50 and 100 μM of the test agents **2** and **3** over a 60 min incubation. The incorporation of $^{14}$C-glycine (53.0 mCi/mmol) into purines /16/ and the incorporation of $^{14}$C-formate (53.0 mCi/mmol) into pyrimidines /17/ was determined in a similar manner.

### Enzyme assays

Studies for the inhibition of various enzyme activities were performed by first preparing the appropriate HL-60 leukemia cell homogenates or subcellular fraction, then adding the drug to be tested during the enzyme assay. For the concentration response studies, inhibition of enzyme activity was determined at 25, 50 and 100 μM of compounds **2** and **3**, after 60 min incubations. DNA polymerase α activity was determined in cytoplasmic isolated extracts [18]. The polymerase activity for α was determined with $^{3}$H-TTP /19/. Messenger-, ribosomal- and transfer-RNA polymerase enzymes were isolated with different concentrations of ammonium sulfate; individual RNA polymerase activities were determined using $^{3}$H-UTP /20.21/. Ribonucleoside reductase activity was measured using $^{14}$C-CDP with dithioerythritol /22/. The deoxyribonucleotides $^{14}$C-dCDP were separated from the ribonucleotides by TLC on PEI plates. Thymidine, TMP and TDP kinase activities were determined using $^{3}$H-thymidine (58.3 mCi/mmol) /23/. Carbamyl phosphate synthetase activity was determined /24/ and citrulline quanitated colorimetrically /25/. Aspartate transcarbamylase activity was measured /24/ and carbamyl aspartate was quantitated colorimetrically /26/. Thymidylate synthetase activity was analyzed by the $^{3}H_2O$ released which was proportional to the amount of TMP formed from $^{3}$H-dUMP /26/. Dihydrofolate reductase activity was determined by a spectrophotometric method /28/. PRPP amidotransferase activity was determined by the method of Spassova *et al.* /29/. IMP dehydrogenase activity was analyzed with 8-$^{14}$C-IMP (54 mCi/mmol) (Amersham, Arlington Heights, IL) after separating XMP on PEI plates (Fisher Scientific) by TLC /30/. Protein content was determined for the enzymatic assays by the Lowry *et al.* technique /31/.

### ct-DNA studies

After deoxyribonucleoside triphosphates were extracted /32/, levels were determined by the method of Hunting and Henderson /33/ with calf thymus DNA, *E. coli* DNA polymerase I, non-limiting amounts of the three deoxyribonucleoside triphosphates not being assayed, and either 0.4 mCi of ($^3$H-methyl)-dTTP or (5-$^3$H)-dCTP. The effects of compounds **2** and **3** on DNA strand scission was determined by the methods of Suzuki *et al.* /34/, Pera *et al.* /35/ and Woynarowski *et al.* /36/. HL-60 leukemia cells were incubated with 10 μCi thymidine /methyl-$^3$H, 84.0 Ci/mmol/ for 24 hr at 37°C. HL-60 cells ($10^7$) were harvested and then centrifuged at 600 g X 10 min in PBS. They were later washed and suspended in 1 ml of PBS. Lysis buffer (0.5 ml; 0.5 M NaOH, 0.02 M EDTA, 0.01% Triton X-100 and 2.5% sucrose) was layered onto a 5-20% alkaline-sucrose gradient (5 ml; 0.3 M NaOH, 0.7 KCl and 0.01 M EDTA); this was followed by 0.2 ml of the cell preparation. After the gradient was incubated for 2.5 hr at room temperature, it was centrifuged at 12,000 RPM at 20°C for 60 min (Beckman rotor SW60). Fractions (0.2 ml) were collected from the bottom of the gradient, neutralized with 0.2 ml of 0.3 N HCl, and measured for radioactivity. Thermal calf thymus DNA denaturation studies, ct-DNA U.V.. absorption studies and DNA viscosity studies were conducted after incubation of compounds **2** and **3** at 100 μM at 37°C for 24 hr /37/ .

### Human DNA Topoisomerase Inhibition

Sample drugs were prepared in DMSO so that the stock final concentration was 5 mM [w/v]. The enzyme assay consisted of test drugs at 50-200 μM, 1 unit of human topoisomerase II (p170 isoform) [TopoGen, Inc., Columbus, OH], ~ 0.5 mg of supercoiled PBR322 DNA in 50 mM Tris buffer, pH 7.5, 15 mM β-mercaptoethanol, 30 mg/ml bovine serum albumin, 1 mM ATP, 10 mM $MgCl_2$ and 150 mM KCl . After 30 min incubation at 37 °C the reaction was terminated with 1% SDS and 1 mg/ml proteinase K (v/v). After an additional hour of incubation, aliquots were applied to a 0.8% agarose TBE gel (v/v) containing 0.5 mg/ml ethidium bromide and 1% SDS (w/v). Following overnight electrophoresis at 30 v (constant), the gel was destained and photographed using a U.V-transilluminator and Polaroid film. Topoisomerase I activity inhibition was assayed by a similar method. The enzyme reaction consisted of test drugs, 0.5 units of human topoisomerase I [TopoGen, Inc., Columbus, OH], 0.5 μg of supercoiled PBR322 DNA in 50 mM Tris-HCl, pH 8.0, 100 mM KCl, 10mM $MgCl_2$, 2 mM 2-mercaptoethanol, 30 μg/ml nuclease-free BSA.

### Statistic Analysis

Data is displayed in tables and figures as the means ± standard deviations of the mean expressed as a percentage of the control value. N is the number of samples per group. The Student's "t"-test was used to determine the probable level of significance (p) between test samples and control samples.

### RESULTS

Compounds **2** and **3** both showed effective cytotoxicity based on $ED_{50}$ values less than 4 μg/ml for L1210, P388, HL-60, Tmolt$_3$, lymphoma HUT-78, HeLa-S$^3$, ileum HCT-8, and liver Hepe-2. Compound 2 had activity against ovary 1-A9, while only compound **3** was only active against prostate PL and glioma UM.

Both compounds were not active against the growth of lung 549, breast MCF-7, osteosarcoma HSO, melanoma SK2, KB nasopharynx, and THP-1 acute monocytic leukemia (Table 1).

Compound **2** was examined for its mode of action in HL-60 leukemia cells (Table 2). DNA and RNA synthesis after 60 minutes was slightly inhibited by 35% and 25% at 100 μM. Protein synthesis after 60 minutes at 100 μM inhibited 55% at 100 μM. Utilization of the DNA template showed that the agent inhibited DNA polymerase α activity by 50% at 100 μM, mRNA polymerase 41%, rRNA polymerase 37%, and tRNA polymerase 52%. A number of enzyme activities were slightly reduced but were not significantly different from the control. Ribonucleotide reductase activity after 60 minutes was inhibited only 12%, while *de novo* purine synthesis was inhibited 18%. Compound **2** mildly suppressed PRPP amido transferase activity at 100 μM by only 6% with an 11% reduction of IMP dehydrogenase activity. Carbamyl phosphate synthase and aspartate transcarbanylase activities were slightly inhibited 14% and 31%. While thymidylate synthase and thymidine kinase activities were increased by 1% and 35%, TMP and TDP kinase was slightly inhibited 22% and 31%. Dihydrofolate reductase activity was markedly inhibited 85%. Studies with ct-DNA showed that compound **2** had no effect on ct-DNA ultraviolet absorption between 220 and 340nm. HL-60 DNA strand scission studies after 24h incubation at 100 μM revealed that compound **2** caused DNA cross-linking (Figure 1). This was consistent with the increase in ct-DNA viscosity after 24 hr at 100 μM. Deoxyribonucleotide levels were all slightly reduced after 60 min incubation at 100 μM.

Compound **3** was also examined for its mode of action in HL-60 leukemia cells (Table 3). DNA and RNA synthesis after 60 minutes was slightly inhibited 35% and 10% at 100μM. Protein synthesis after 60 minutes at 100 μM was inhibited 48% at 100μM. Utilization of the DNA template was moderately inhibited at 100 μM with inhibition of DNA polymerase α activity 20%, mRNA polymerase activity 44%, rRNA polymerase activity 40%, and tRNA polymerase activity 39%. Ribonucleotide reductase activity was inhibited only 25%, while *de novo* purine synthesis was inhibited 35% after 60 min. Compound **3** mildly suppressed PRPP amido

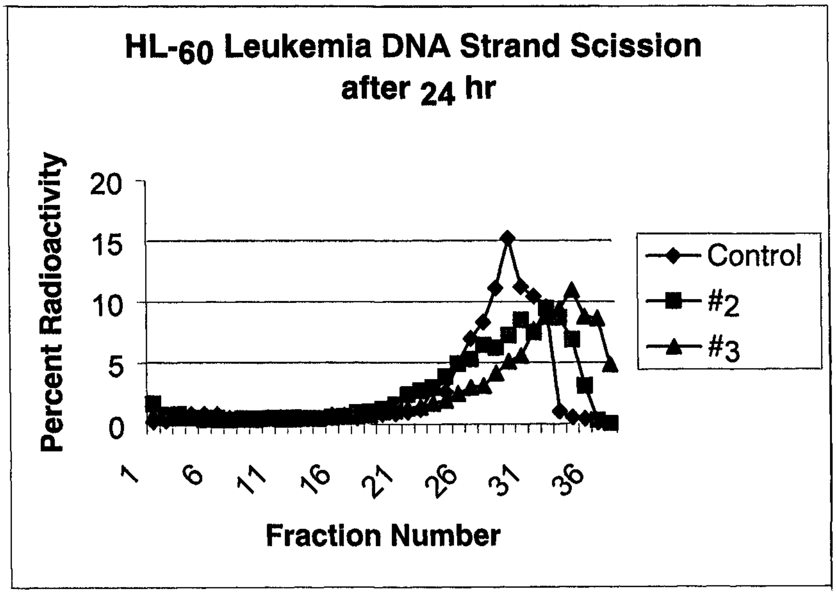

**Fig. 1:** DNA Strand Scission by the Cyanoboranes after 24 hr at 100 mM

**Table 1**

Cytotoxicity of Cyanoborane Compounds

| **Tumor Cell Line** | **2** | **3** | **6-MP** | **Ara-C** | **VP-16 Etoposide** | **5-FU** |
|---|---|---|---|---|---|---|
| L1210 mouse leukemia | 2.96 | 3.56 | 2.43 | 3.07 | 1.83 | 1.41 |
| P388 mouse lymphocytic leukemia | 2.58 | 1.31 | 2.04 | 0.79 | 0.99 | 1.41 |
| HL-60 human Leukemia | 3.53 | 2.62 | 3.35 | 4.00 | 4.43 | 5.28 |
| Tmolt$_3$ T cell Leukemia | 3.17 | 2.17 | 1.62 | 2.67 | 1.00 | 2.14 |
| T molt$_4$ T cell Leukemia | 4.24 | 4.56 | 2.67 | 2.36 | 1.92 | 2.75 |
| HuT-78 Lymphoma | 3.53 | 2.96 | 1.68 | 2.50 | 1.33 | 5.81 |
| THP-1 Acute Monocytic Leukemia | 6.73 | 7.11 | 3.03 | 2.54 | 3.27 | 1.12 |
| HeLa-S$^3$ susp Uterine | 2.75 | 3.77 | 2.12 | 2.13 | 1.69 | 2.47 |
| KB Nasopharynx | 4.88 | 5.74 | 11.04 | 2.84 | 3.32 | 1.25 |
| Lung A- 549 | 7.69 | 4.05 | 4.71 | 5.62 | 4.74 | 3.58 |
| Liver Hepe-2 | 3.20 | 2.69 | | | | |
| Ovary 1-A9 | 2.63 | 4.47 | 6.64 | 5.39 | 6.24 | |
| Breast MCF-7 | 6.07 | 6.73 | 8.84 | 12.45 | 11.00 | 6.82 |
| Glioma UM 86 | 5.98 | 3.68 | 4.46 | 1.88 | 2.44 | 1.28 |
| Ileum HCT-8 | 1.93 | 3.97 | 1.15 | 2.54 | 1.13 | 1.30 |
| Prostate PL | 4.63 | 2.98 | | | | |
| Osteosarcoma HSO | 6.46 | 8.79 | 9.13 | 0.86 | 3.57 | 8.73 |
| Melanoma SK2 | 6.37 | 7.51 | 6.86 | 10.53 | 3.53 | 5.93 |
| Normal RMPI 1788 | 9.14 | 8.41 | -- | -- | -- | |

$ED_{50}$ values $\geq 4$ µg/ml are required for significant activity

**Table 2**

Effects of Compound 1 HL60 cell metabolism after 60 min incubation.

| | Percent of Control ( $\bar{X} \pm$ S.D.) | | | |
|---|---|---|---|---|
| Assay (N = 6) | Control | 25 μM | 50 μM | 100 μM |
| DNA Synthesis | 100 ± 6[a] | 81± 4 | 68±5* | 65±4* |
| RNA Synthesis | 100 ± 5[b] | 98±5 | 94±4 | 75±4* |
| Protein Synthesis | 100 ± 6[c] | 55±4* | 45±5* | 45±3* |
| DNA Polymerase α | 100 ± 5[d] | 76±4* | 72±5* | 50±4* |
| mRNA Polymerase | 100 ± 4[e] | 71±5* | 59±4* | 59±3* |
| rRNA Polymerase | 100 ± 6[f] | 69±6* | 65±5* | 63±5* |
| tRNA Polymerase | 100 ± 6[g] | 57±5* | 56±6* | 48±5* |
| Ribonucleotide Reductase | 100 ± 6[h] | 94±6 | 92±5 | 88±4 |
| *De Novo* Purine Synthesis | 100 ± 6[i] | 110±6 | 86±5 | 82±4 |
| PRPP Amido Transferase | 100 ± 6[j] | 100±7 | 95±5 | 94±4 |
| IMP Dehydrogenase | 100 ± 7[k] | 96±5 | 94±6 | 89±5 |
| *De Novo* Pyrimidine Synthesis | 100 ± 6[l] | 101±5 | 91±5 | 76±4* |
| Carbamyl Phosphate Synthetase | 100 ± 6[m] | 89±5 | 89±5 | 86±6 |
| Aspartate Transcarbamylase | 100 ± 7[n] | 101±5 | 100±6 | 69±4* |
| Thymidylate Synthase | 100 ± 6[o] | 128±5* | 128±6* | 101±5 |
| Thymidine Kinase | 100 ± 5[p] | 178±7* | 135±5* | 135±6* |
| TMP kinase | 100 ± 4[q] | 104±5 | 90±4 | 78±3* |
| TDP Kinase | 100 ± 4[r] | 89±5 | 81±4* | 69±3* |
| Dihydrofolate Reductase | 100 ± 6[s] | 49±4* | 32±3* | 15± 2* |
| d(ATP) | 100 ± 4[t] | | | 87±5 |
| d(GTP) | 100 ± 6[u] | | | 75±4* |
| d(CTP) | 100 ± 6[v] | | | 89±5 |
| d(TTP) | 100 ± 6[w] | | | 83±5 |

* $P \leq 0.001$;

[a] 45011 dpm [f] 8394 dpm [k] 4658 dpm [p] 1511 dpm [u] 11.21 pmoles

[b] 4226 dpm [g] 5151 dpm [l] 7316 dpm [q] 320 dpm [v] 13.65 pmoles

[c] 5343 dpm [h] 63565 dpm [m] 1.242 μmoles citrulline [r] 286 dpm [w] 16.73 pmoles

[d] 7125 dpm [i] 17646 dpm [n] 1.030 mol N-carbamyl aspartate [s] 0.092 OD units

[e] 5693 dpm [j] 0.164 OD units [o] 13890 dpm [t] 9.02 pmoles

**Table 3**

Effects of Compound **2** HL-60 Leukemia cell metabolism after 60 min incubation.

Percent of Control ( $\bar{X} \pm$ S.D.)

| Assay (N = 6) | Control | 25 μM | 50 μM | 100 μM |
|---|---|---|---|---|
| DNA Synthesis | $100 \pm 6^{a}$ | 66±4* | 65±3* | 65±4* |
| RNA Synthesis | $100 \pm 5^{b}$ | 97±5 | 94±6 | 90±4 |
| Protein Synthesis | $100 \pm 6^{c}$ | 60±4* | 56±4* | 52±3* |
| DNA Polymerase α | $100 \pm 5^{d}$ | 94±5 | 93±5 | 80±4* |
| mRNA Polymerase | $100 \pm 4^{e}$ | 82±5 | 67±4* | 56±4* |
| rRNA Polymerase | $100 \pm 6^{f}$ | 86±6 | 71±5* | 60±4* |
| tRNA Polymerase | $100 \pm 6^{g}$ | 74±5 | 63±4* | 61±3* |
| Ribonucleotide Reductase | $100 \pm 6^{h}$ | 102±6 | 88±5 | 85±5 |
| *De Novo* Purine Synthesis | $100 \pm 6^{i}$ | 76±4* | 66±4* | 65±4* |
| PRPP Amido Transferase | $100 \pm 6^{j}$ | 92±5 | 89±5 | 88±6 |
| IMP Dehydrogenase | $100 \pm 7^{k}$ | 101±6 | 93±5 | 85±5 |
| *De Novo* Pyrimidine Synthesis | $100 \pm 6^{l}$ | 93±4 | 80±4* | 79±3* |
| Carbamyl Phosphate Synthetase | $100 \pm 6^{m}$ | 116±5 | 104±5 | 102±6 |
| Aspartate Transcarbamylase | $100 \pm 7^{n}$ | 104±6 | 98±4 | 58±4* |
| Thymidylate Synthase | $100 \pm 6^{o}$ | 101±5 | 99±5 | 24±3* |
| Thymidine Kinase | $100 \pm 5^{p}$ | 169±8* | 152±6* | 72±4* |
| TDP kinase | $100 \pm 4^{q}$ | 96±5 | 74±4* | 57±3* |
| TTP Kinase | $100 \pm 4^{r}$ | 103±5 | 101±4 | 54±4* |
| Dihydrofolate Reductase | $100 \pm 6^{s}$ | 57±4* | 40±3* | 37±3* |
| d(ATP) | $100 \pm 4^{t}$ | | | 88+5 |
| d(GTP) | $100 \pm 6^{u}$ | | | 89+5 |
| d(CTP) | $100 \pm 6^{v}$ | | | 88±5 |
| d(TTP) | $100 \pm 6^{w}$ | | | 75±5* |

* $P \leq 0.001$; transferase activity at 100 μM by only 12% with a 15% reduction of IMP dehydrogenase activity. Carbamyl phosphate synthase activity showed an increase of 14%, while aspartate transcarbanylase activity was inhibited 42%. Only thymidylate synthase activity was markedly suppressed 76%, with thymidine kinase activity marginally inhibited 28%, TMP kinase activity 43% and TDP kinase activity 46%. Dihydrofolate reductase activity was suppressed 63%. Studies with ct-DNA showed that compound **3** had no effect on ct-DNA ultraviolet absorption between 220 and 340nm. HL-60 DNA strand scission after 24h incubation at 100

μM revealed that compound **3** caused DNA cross linking (Figure 1) which was consistent with the observed increased in ct-DNA viscosity after 24 hr at 100 μM. Deoxyribonucleotide pools were slightly reduced after 60 min incubation with agents at 100 μM. Human topoisomerase I and II activity was not inhibited by compounds 2 or 3 at 100 μM.

### DISCUSSION

$N^6$-Benzoyladenine-cyanoborane (**2**), and 6-triphenylphosphonylpurine-cyanoborane (**3**) proved to be cytotoxic in suspended cancer cells. Surprisingly these compounds were also cytotoxic in solid liver Hepe-2 and ileum HCT-8 carcinoma. In mode of action studies in human leukemic HL-60 cells, both compounds demonstrated inhibition of DNA and protein syntheses after 60 min at 100 μM. These compounds inhibited RNA synthesis to a lesser extent. The utilization of the DNA template was suppressed by the compounds as determined by inhibition of the activities of DNA polymerase α, m-RNA polymerase, r-RNA polymerase and t-RNA ploymerase which would cause adequate inhibition of the synthesis of both DNA and RNA. Because the d[NTP] pool levels were slightly reduced after 60 min further inhibition of DNA synthesis would occur. Both compounds remarkably inhibited dihydrofolate reductase activity, especially compound **2**. This would cause the reduction of the one carbon transfer for purine and pyrimidine syntheses /2/. However, the *de novo* synthesis of purine and pyrimidines was only marginally affected by the compounds as were their regulatory enzyme activities /2/. Ribonucleotide reductase activity was moderately inhibited which would reduce the amount of ribonucleotide converted to deoxyribonucleotides for DNA synthesis. The reduction of TMP and TDP kinase activities would further reduced thymidine nucleotides levels demonstrated significantly by compound **3**. Both compounds appeared to have caused cross-linking of the DNA strands after 24 hr at 100 μM in HL-60 cells, which was consistent with the observed increased in ct-DNA viscosity after 24 hr at 100 μM and lack of inhibition of DNA topoisomerase I and II activities with no DNA-protein linked breaks. Neither compounds interacted with the DNA molecule itself through alkylation of the nucleotide bases nor caused DNA interculation between base pairs.

Previously studied thymidine, inosine, cytidine, guanosine, and arbinoside cyanborane nucleotides have demonstrated a similar pattern of cytotoxicity on the growth of suspended murine and human tumor cells and solid human tumors. Those nucleoside and nucleotide cyanboranes inhibited DNA and protein synthesis, with a select few of the derivatives reducing RNA synthesis after 1 hr /2/. Mutliple targets of the cyanboranes in DNA synthesis were demonstrated by the compounds. For the nucleoside cyanboranes the major sites of inhibition were IMP dehydrogenase and PRPP amido tranferase activities, suppressing *de novo* purine synthesis of $Tmolt_3$ leukemia cells /2/. In contrast, the *de novo* synthesis of purine, pyrimidine and their regulatory enzyme activities were only marginally suppressed by the current compounds. Although similar nucleoside cyanoboranes inhibited dihydrofolate reductase activity, the current compounds were more potent. The boranated nucleosides cause a reduction of thymidylate synthase activity whereas only compound **3** decreased activity while compound **2** increased activity. However, both types of compounds inhibited TMP and TDP kinase activity and marginally reduced d[NTP] pools. Some of the nucleoside cyanoboranes caused DNA strand scission [thymidine] whereas others [ ribose and arabinoside] caused DNA cross-linking as the current compounds. However, none of the cyanboranes targeted the DNA molecule itself.

### CONCLUSION

$N^6$-Benzoyladenine-cyanoborane (**2**) and 6-triphenylphosphonylpurine-cyanoborane (**3**) have been proven to be effective antineoplastic agents in their overall reduction of DNA and protein replication in respect to killing cancer cells. The inhibition of dihydrofolate reductase activity and/or thymidylate synthetase adds to the overall inhibition of DNA and protein synthesis. Even though both compounds showed DNA cross-linking, neither compound interacted with the DNA molecule itself through alkylation of the nucleotide bases nor caused DNA intercalation between base pairs. Sufficient activity was demonstrated by these cyanoborane derivatives to warrant further investigation as potential antineoplastic for clinical use.

### ACKNOWLEDGEMENT

The authors wish to thank the NSF NMR Collaborative training partnership Grant DUE 995269 [Rider] and the National Leukemic Research Association [IHH].

### REFERENCES

1. A. Sood, B.F. Spielvogel, W.J. Powell, K.F. Bastow, M.C. Miller and I.H. Hall, Cytotoxicity of ribo- and arabinoside boron nucleosides in tissue culture cells, *Anticancer Research* **14**, 1483-1488 (1994).
2. I.H. Hall, E.S. Hall, L.K. Chi, B.R. Shaw, A. Sood and B.F. Spielvogel, Antineoplastic activity of boron-containing thymidine nucleosides in $Tmolt_3$ leukemia cells, *Anticancer Research* **12**, 1091-1908 (1992).
3. B.S. Burnham, S.Y. Chen, A. Sood, B.F. Spielvogel, M.C. Miller and I.H. Hall, The cytotoxicity of 3'-aminocyanoborane-2', 3'-dideoxypyrimidines in murine and human tissue cultured cell lines, *Anticancer Research* **15**, 951-958 (1995).
4. I.H. Hall, A.L. Elkins, J. Tomasz and B.F. Spielvogel, The cytotoxicity of adenosine 5'[N, N-di-(γ-o-carboranyl)propyl phosphorodiamidate in human $Tmolt_3$ leukemia cells. *Anticancer Research* **17**, 151-156 (1997).
5. I.H. Hall, B.S. Burnham, A.L. Elkins, A. Sood, W. Powell, J. Tomasz and B.F. Spielvogel, Boronated pyrimidines and purines as cytotoxic, hypolipidemic and anti-inflammatory agents. *Metal-Based Drugs* **3**, No. 3 (1996).
6. A. Sood, B.R. Shaw, B.F. Spielvogel, E.S. Hall, L.K. Chi and I.H. Hall, The synthesis and anti-neoplastic activity of $N^2$-isobutyryl-2'-deoxyguanosine-$N^7$-cyanoborane derivatives. *Phamazie* **47**, H. 11 (1992).
7. I.H. Hall, A.L. Elkins, A. Sood, J. Tomasz and B.F. Spielvogel, Boron substituted deoxyribonucleosides as cytotoxic agents. *Anticancer Research* **16**, 3709-3714 (1996).
8. A. Sood, B.F. Spielvogel, B.R. Shaw, L.D. Carlton, B.S. Burnham, E.S. Hall and I.H. Hall, The synthesis and antineoplastic activity of 2'-deoxynucloside-cyanoboranes in murine and human culture cells. *Anticancer Research* **12**, 335-344 (1992).

9. C. Sood, A. Sood, B.F. Spielvogel, J. Yousef, B.S. Burnham and I. Hall, Synthesis and antineoplastic activity of some cyano-, carboxy-, carbomethoxy-, and carbamoylborane adducts of heterocyclic amines, *J. Pharm. Sci.*, **80**, 1133-1140 (1991).
10. M.K. Das and S. Roy, Convenient and high-yield synthesis of triphenylphosphine-cyanoborane and -dihaloboranes, *Synth. React. Inorg. Met.-Org. Chem.* **15**, 53-59 (1985).
11. A.J. Gordon and R.A. Ford, *The Chemist's Companion, A Handbook of Practical Data, Techniques, and References*. New York, John Wiley and Sons, 1972; pp.429-436.
12. Only a portion of the reaction mixture adsorbed on silica gel was purified.
13. R.J. Geran, N.H. Greenburg, M.M. MacDonald, A.M. Schumacher and B.J. Abbott, Protocols for screening chemical agents and natural products against animal tumors and other biological systems. *Cancer Chemo Rep* **3**, 9-11(1972).
14. A.L. Leibovitz, J.C. Stinson, W.B. McComb III, C.E. McCoy, K.C. Mazur and N.D. Mabry, Classification of human colorectal adenocarcinoma cell lines. *Cancer Res* **36**, 4562-4569 (1976).
15. L.L. Liao, S.M. Kupchan and S.B. Horwitz, Mode of action of the antitumor compound bruceatin, an inhibitor of protein synthesis. *Mol Pharmacol* **12**, 167-176 (1976).
16. E. Cadman, R. Heimer and C. Benz, The influence of methotrexate pretreatment on 5-fluorouracil metabolism in $L_{1210}$ cells. *J Biol Chem* **256**:1695-1704 (1981).
17. R.I. Christopherson, M.L. Yu and M.E. Jones, An overall radioassay for the first three reactions of *de novo* pyrimidine synthesis. *Anal* Bioc*hem* **11**, 240-249 (1981).
18. H. Sawada, K. Tatsumi, M. Sadada, S. Shirakawa, T. Nakamura and G. Wakisaka, Effects of neocarzinostatin on DNA synthesis in $L_{1210}$ cells. *Cancer Res* **34**, 3341-3346 (1974).
19. D.C. Eichler, P.A. Fisher and D. Korn, Effect of calcium on the recovery distribution of DNA polymerase from cultured human cells. *J Biol Chem* **252**, 4011-4014 (1977).
20. K.M. Anderson, I.S. Mendelson and G. Guzik, Solubilized DNA-dependent nuclear RNA polymerases from the mammary glands of late-pregnant rats. *Biochem Biophys Acta* **383**, 56-66 (1975).
21. I.H. Hall, G.L. Carlson, G.S. Abernathy and C. Piantadosi, Cycloalkanones IV. Antifertility Agents. *J Med Chem* **17**, 1253-1257(1974).
22. E.C. Moore and R.B. Hurlbert, Regulation of mammalian deoxyribonucletide biosynthesis by nucleotide or activators and inhibitors. *J Biol Chem* **241**, 4802-4809 (1966).
23. F. Maley and S. Ochoa, Enzymatic phosphorylation of deoxycytidylic acid. *J Biol Chem* **233**, 1538 - 1543 (1958).
24. S.M. Kalman, P.H. Duffield and T.J. Brzozwski, Purification and properties of a bacterial carbamyl phosphate synthetase. *J Biol Chem* **241**, 1871-1877 (1966).
25. R.M. Archibald, Determination of citrulline and allontoin and demonstration of citrulline in blood plasma. *J Biol Chem* **156**, 121-142 (1944).
26. S.B. Koritz and P.P. Gohen, Colorimetic determination of carbamyl amino acid and related compounds. J Biol Chem **209**:145-150 (1954).
27. A. Kampf, R.L. Barfknecht, P.J. Schaffer, S. Osaki and M.P. Mertes, Synthetic inhibitors of Escherichia Coli calf thymus and Ehrlich ascites tumor thymidylate synthetase. *J Med Chem* **19**, 903-908 (1976).

28. Y.K. Ho, T. Hakala and S.F. Zakrzewski, 5-(1-Adamantyl) pyrimidines as inhibitors of folate metabolism. *Cancer Res* **32**, 1023-1028 (1971).
29. M.K. Spassovaa, G.C. Russev and E.V. Goovinsky, Some pyrazoles as inhibitors of purine biosynthesis *de novo*. *Biochem Pharmacol* **25**, 923 -924 (1976).
30. J.H. Becker and G.W. Lohr, Inosine-5'-phosphate dehydrogenase activity in normal and leukemic blood cells. *Klin Wochenschr* **57**, 1109-1115 (1979).
31. O.H. Lowry, J. Rosebrough, A.L. Farr and R.J. Randall, Protein measurement with folin phenol reagent. *J Biol Chem* **193**, 265-275 (1951).
32. A.S. Bagnara and L.R. Finch, Quantitative extraction and estimation of intracellular nucleotide-triphosphate in Escherichia Coli. *Anal Biochem* **45**, 24-34 (1971).
33. D. Hunting and J.F. Henderson, Determination of deoxyribonucleoside triphosphates using DNA polymerase, a critical evaluation. *Can J Biochem* **59**, 723-727 (1982).
34. H. Suzuki, T. Nishimura, S.K. Muto and N. Tanaka, Mechanism of action of macromomycin, DNA strand scission, inhibition of DNA synthesis mitosis. *J Antibacteriol* **32**, 875-883 (1978).
35. J.F. Pera, Sr., C.J. Rawlings, J. Shackleton and J.J. Roberts, Quantitative aspects of the formation and loss of DNA. *Biochem Biophys Acta* **655**, 152-166 (1981).
36. J.W. Woynarowski, T.A. Beerman and J. Konopa, Introduction of deoxyribonucleic acid damage in HeLa $S^3$ cells by cytotoxic and antitumor sesquiterpine lactones. *Biochem Pharmacol* **30**, 3005-3007(1981).
37. Y. Zhao, I.H. Hall, C.B. Oswald, T. Yokoi and K.H. Lee, Anti-malarial agents III. Mechanism of action of artesunate against *Plasmodium Berghi* infection. *Chem Phar Bull* **35**, 2052-2061 (1987).

